# Residential Links to Air Pollution and School Children with Asthma in Vilnius (Population Study)

**DOI:** 10.3390/medicina56070346

**Published:** 2020-07-13

**Authors:** Sarunas Alasauskas, Ruta Ustinaviciene, Mindaugas Kavaliauskas

**Affiliations:** 1 Department of Environmental and Occupational Medicine, Lithuanian University of Health Sciences, 47181 Kaunas, Lithuania; ruta.ustinaviciene@lsmuni.lt; 2 Department of Applied Mathematics, Kaunas University of Technology, 51368 Kaunas, Lithuania; m.kavaliauskas@ktu.lt

**Keywords:** air pollution, residential exposure, asthma, children, GIS, logistic regression

## Abstract

*Background and objectives:* Many studies have been carried out on the negative health effects of exposure to PM_10_, PM _2.5_, NO_2_, CO, SO_2_ and B[a]P for small populations. The main purpose of this study was to explore the association of air pollution to diagnosis of asthma for the whole huge population of school children between 7–17 years in Vilnius (Lithuania) using geographical information system analysis tools. *Material and Methods:* In the research, a child population of 51,235 individuals was involved. From this large database, we identified children who had asthma diagnosis J45 (ICD-10 AM). Residential pollution concentrations and proximity to roads and green spaces were obtained using the ArcGIS spatial analysis tool from simulated air pollution maps. Multiple stepwise logistic regression was used to explore the relation between air pollution concentration and proximity between the roads and green spaces where children with asthma were living. Further, we explored the interaction between variables. *Results:* From 51,235 school children aged 7–17 years, 3065 children had asthma in 2017. We investigated significant associations, such as the likelihood of getting sick with age (odds ratio (OR) = 0.949, *p* < 0.001), gender (OR = 1.357, *p* = 0.003), NO_2_ (OR = 1.013, *p* = 0.019), distance from the green spaces (OR = 1.327, *p* = 0.013) and interactions of age × gender (OR = 1.024, *p* = 0.051). The influence of gender on disease is partly explained by different age dependency slopes for boys and girls. *Conclusions:* According to our results, younger children are more likely to get sick, more cases appended on the lowest age group from 7 to 10 years (almost half cases (49.2%)) and asthma was respectively nearly twice more common in boys (64.1%) than in girls (35.9%). The risk of asthma is related to a higher concentration of NO_2_ and residence proximity to green spaces.

## 1. Introduction

Scientific data show that exposure to pollutants in childhood can increase the future risk of asthma and lead to lifelong consequences [1]. Children are more vulnerable, and that is no longer debatable, but knowledge about disease-causing factors still is controversial. Environmental factors, both individually and in combination with genetic factors, are considered to be major determinants [2]. We are providing a full population design study by individuals to find interactions between pollutants and other variables with the purpose to create an asthma risk model for 7–17 years school children. We know that pediatric airways are smaller in diameter and shorter in length than in adults, so the impact of exposure to air pollutants can be greater, and result in respiratory problems including asthma. The delivery of pollutants to the respiratory tract of infants and children differs from adults, not only by the amount of pollutants, but also by their localization [3]. Children may also be more vulnerable to air pollution than adults because they play closer to the ground, spend more time outdoors, breathe faster, have higher physical activity (which causes inhalation of more pollutants) and since also its effects are related to lower body weight or overweight [4,5]. 

Previous studies have also addressed gender aspects, whether asthma or wheeze affects boys more than girls, and it depends on the growth of airway size and immunological distinction, but the influence of air pollution on respiratory diseases by gender is still under discussion [6]. Some studies have indicated that only a potential relationship might exist and that the links established are poor. The design of the studies and exposure assessment are very different and need additional evaluation. 

It is clear that the increase in asthma cases cannot be determined by genetic factors alone [7]. It is necessary to investigate the effect on asthma from different pollutants in one study, also to underline green spaces’ possible positive effect for the whole population and combine it with gender and age aspects.

Asthma in children has been on the rise in recent decades, and despite significant improvements in fuel quality and engine technology, vehicle traffic emissions have become a major pollutant, mainly in urban areas. In Europe, exhaust fumes from vehicles are considered to be the most important sources of nitrogen oxides (NO), carbon monoxide (CO), volatile organic compounds (VOCs), B[a]P (Benzo[a]pyrene) and particulate matter (PM_10_, PM_2.5_) pollution [8]. Many researchers have related living near traffic or polluted areas with asthma and other respiratory symptoms [9].

Traffic pollution in big urban areas and close to main roads is prevalent, but the concentrations of the pollutants are different because they are influenced by multiple meteorological or environmental features: topography, terrain, green spaces, city plan or traffic organization [10]. Green spaces filled with trees and shrubs play an important role in air contamination prevention. Trees remove a large amount of air pollutants and the air becomes cleaner and safer for the living environment [11,12]. Some studies, due to living distance near green spaces, identify the negative effect on asthma [13]. Positive effects may be associated with lower air pollution in green areas, and negative effects may be associated with exposure to pollen for allergic respiratory diseases [14,15]. One study of a cohort of seven children found that children living within 500 m of green spaces were at higher risk of developing allergic diseases [16]. 

NO_2_ is the main marker of traffic-related pollution with its increased levels affecting a higher number of children, reducing lung function [17]. Further, associations between increased exposure to PM_10_, PM_2.5_, sulphur dioxide (SO_2_), CO, ozone (O_3_), B[a]P and the risk of asthma to children were found [18,19,20,21,22,23]. Long exposure to air pollution has been associated with chronic respiratory disease [24,25,26,27].

Vilnius is the capital, the biggest and most polluted city in Lithuania, moreover, the number of children with asthma increases every year. The main purpose of our study was to evaluate the effect of the spatial distribution of pollutant concentrations, distance from major roads, the distance from green spaces to living places and individual factors on the risk of asthma in all school children of ages 7–17 years living in Vilnius for an entire population. All school children are exploratory included in the GIS database and other statistical analysis. The cases are medically confirmed, not produced from a survey.

## 2. Materials and Methods

The capital of Lithuania, Vilnius, is not very dense. In one square kilometer lives 1382 people. In Vilnius, green spaces cover about 40% of all territory, which is substantial. A big role in air pollution in the city is played by the significant number of vehicles: we had about 546,382 citizens using 192,086 vehicles in 2017. As a result, we are using one car for 2.8 people. According to air pollution records in 2017, Vilnius did not exceed air quality limits except for Benzo(a)pyrene.

A cross-sectional study was conducted and included all 51,235 school children between 7–17-years-old living in Vilnius. There was one specific exclusion criterion, that is, we deleted 22 children from database who were registered to the same address. The address was established for political and financial reasons, that people could declare their living place if they do not have their own and pay taxes. From the entire school child population, we observed 3065 children with asthma diagnosis J45 (ICD-10 AM) in the year 2017. Boys accounted for 51 percent of the entire child population, girls 49 percent. As the age increased, the proportion of children decreased steadily, from 12.4 to 7.9% at 7 and 17 years, respectively. There is a more detailed sample description in the section of results. All depersonalized cases of asthma, demographic and location data for the children were derived from the Children’s Health Monitoring Information System (CHMIS) database. CHMIS is a nationwide database created to provide clear statistics about health and risk factors, to monitor children’s public health inequalities in the country, and to provide recommendations for school public health specialists in the education process. It collects data from 8 different national registers of Lithuania: population register, student register, address register, compulsory health insurance information system, social services information system, deaths and their causes register, E. health services and cooperation infrastructure information system. 

Air pollution was modeled by the Environmental Protection Agency using “ADMS-Urban 4.1.1 software Maps” (ADMS) and official data. This software is recommended by the government for air pollution modeling. Official models of air pollutants PM_10_, PM_2.5_, NO_2_, SO_2_, and B[a]P ambient diffusion in Vilnius were created using Gaussian spreading algorithms. The model included the following data: physical parameters (velocity, height) of all stationary air pollution sources and quantities of pollutant emissions into the ambient air during the reporting year, hourly information of meteorological stations (temperature, wind speed, wind direction, cloudiness, precipitation), average annual traffic data (number of trucks and cars per hour, average speed, road width, canyon), the influence of airport pollution, number of dwellings, type of heating and types of fuel used for heating (gas, wood, centralized heating). In the preparation of maps for the modeling and diffusion of ambient air pollution, all the above-mentioned data, together with the city relief layer and background concentrations of relatively clean rural areas, were brought into the model [28]. After the initial modeling, the data (i.e., concentrations) were compared with the actual measurements of the 4 automatic air pollution stations, with a correct pollution measurement buffer of about 2 km. Very different data were adjusted on the basis of GIS. According to a European Union directive, the modeled pollutant concentrations must not differ by more than 30%. These models comply with the EU directives and are used to analyze national and local air pollution problems and inform the general public and official bodies.

The pollution concentration data for individuals were extracted using the ArcGIS spatial analysis tool, from modeled air pollution maps. We used the same tool we to calculate distance from main roads (A, B, C category roads, which are the most intensive transport “arteries”), green spaces (areas with trees and bushes) and living places (geocoded residency address). We used air pollution raster maps, which were divided into cells (pixels) with an approximate size of 147 × 147 m. We combined the GIS layers of all children living places (XY points), sick children (XY points) and raster pollution maps. Then, from each cell, we extracted different pollution concentrations for children living places.

**Figure 1:**
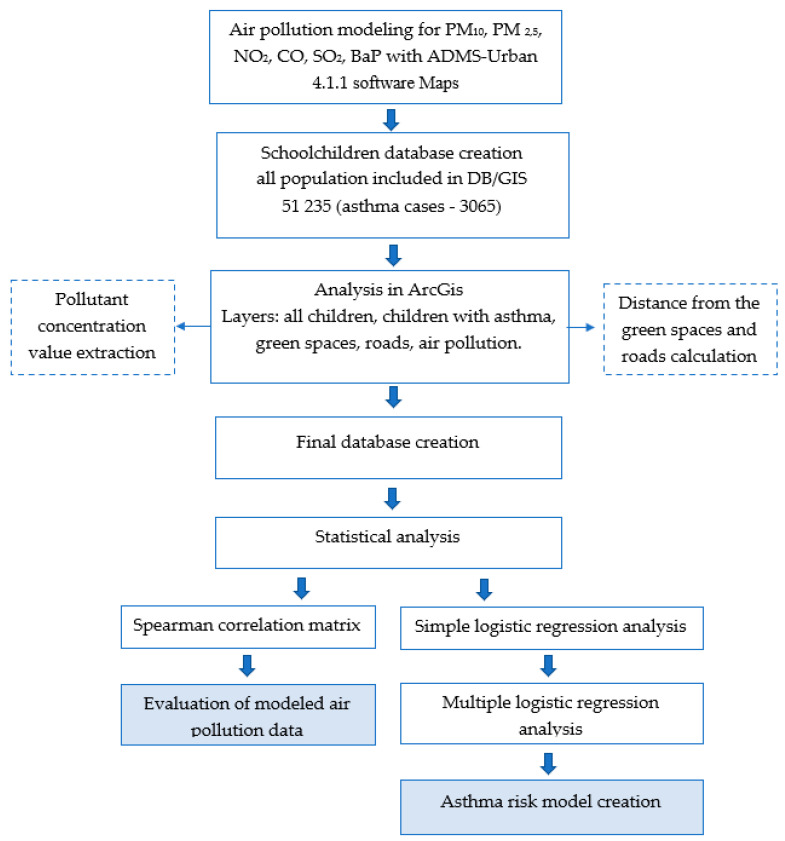
Flowchart of the study.

We used the Spearman correlation coefficient to evaluate modeled data, the relationship between the pollutants and the distance from roads and green spaces. The association between exposure, distance from the main roads and asthma cases were analyzed using multiple logistic regression with the R and SPSS statistical software. At first, we used a simple logistic regression and explored dependencies of asthma probability on every single variable, one-by-one. After this, only significant variables from simple logistic regression models were included in a further modeling process. We used stepwise forward and backward procedures based on the Akaike information criterion (AIC), for variable selection, and finally, after this, we created a multiple logistic regression model with significant factors which may affect the onset of asthma. Neither stepwise forward nor stepwise backward methods ensure finding the optimal model. The authors attempted to use both of these methods to maximize the chances of finding the optimal model. If different models were obtained, the one with the lower AIC value was considered the final model. Both stepwise forward and backward selected the same model in the logistic regression models without interaction. If pairwise interactions were included, different models were obtained. The model obtained by the stepwise forward method was superior with respect to AIC. In Figure 1, you can look at the study flowchart.

The study was conducted with the approval of the Vilnius Regional Bioethical Committee, No. 158200-18/3-1016-514.

## 3. Results

**Figure 2:**
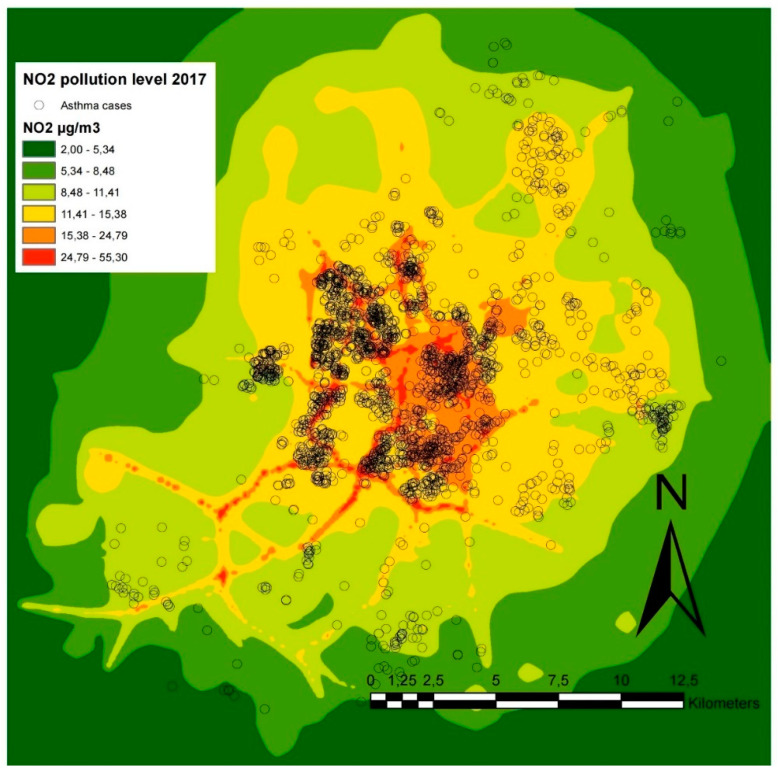
Map with the spatial distribution of children with asthma cases in Vilnius with NO_2_ pollution data.

From 51,235 school children between 7–17-years-old, we found 3065 children with asthma J45 (ICD-10 AM) cases in the 2017 year. Most cases were found in densely populated and polluted neighborhoods, due to the developed infrastructure illustrated in Figure 2.

Air pollution in Vilnius is monitored via four automatic air quality measurement stations. According to air pollution records from 2017, Vilnius did not exceed air quality limits except for Benzo(a)pyrene. Annual air pollution concentrations for the 2017 year and the quality limits are presented in Table 1.

Demographic statistics for school children are presented in Table 2. More cases appended on the lowest age group from 7 to 10 years: almost half the cases (49.2%). Asthma was respectively nearly twice as common in boys (64.1%) than in girls (35.9%). 

The analysis of correlation allowed us to see the links between the pollutants and their possible sources. Spearman’s correlation coefficient reliability between variables was with *p* values < 0.01. As shown in Table 3, there is evidence of a very strong correlation between PM_10_ and PM_2.5_ (*r* = 0.97), strong correlations between NO_2_ and CO (*r* = 0.85), and B[a]P with PM_10_, PM_2.5_ (*r* = 0.74), NO_2_ (*r* = 0.73). Moderate correlations between PM_10_, PM_2.5_ with NO_2_ (*r* = 0.51), CO (*r* = 0.53), also B[a]P with CO (*r* = 0.65). Correlations between proximity variables and air pollutants were negative and weak with NO2(*r* = −0.28), CO (*r* = −0.30) and B[a]p (*r* = −0.29), meaning that as the distance to the road decreases, the concentration increases. Distance from green spaces showed a positive weak correlation between all pollutants, confirming a lower concentration of pollution in a greener neighborhood.

To identify the impact of air pollution and other variables on asthma, firstly, we used a simple logistic regression separated for each variable. The results are presented in Table 4. We found significant association between age, gender, PM_10_, PM_2.5_, NO_2_, B[a]P and green spaces. We did not find a significant association between SO_2_, CO and distance from the main intensive roads.

In the multiple logistic regression model, we included only significant variables from Table 4. For better model creation, we used stepwise forward and backward procedures for selecting variables based on the AIC. After it, from the model, insignificant variables (PM_10_ (OR = 1.0002, *p* = 0.860), PM_2.5_ (OR = 1.0001, *p* = 0.925), B[a]P (OR = 1.006, *p* = 0.815)) were excluded and, in the end, we found that age plays the biggest role: younger children are more likely to get sick. Boys are more susceptible to asthma than girls. The biggest roles are played by pollution with NO_2_ and proximity between the green spaces and the residence of the children (Table 5). 

We also built a model with pairwise interactions of all candidate variables significant from Table 4. We used stepwise forward and backward procedures based on AIC. In this case, these two procedures obtained different models. The model selected by the stepwise forward procedure was selected as a final model based on the value of the Akaike information criterion. Parameters of the model are presented in Table 6.

The only interaction included into the model was the interaction between age and gender. The *p*-value of this interaction is a little above the significance level, but it was included into the model by the stepwise procedure based on AIC. Both age and gender had a high statistical significance (*p*-value < 0.001) in the model without interaction terms. Since the sample size is large (*n* = 51,235), the model is capable to detect the small interactions between statistically significant variables. Therefore, the inclusion of an age and gender interaction is not unexpected.

Gender is a binary variable. This allows to provide another interpretation of the result. The age coefficient β is equal −0.052 (OR = 0.949) for girls (Gender = 0), but for boys (Gender = 1) it adds up to the coefficient of the interaction. Therefore, the total value of the age coefficient for boys β= −0.052 + 0.02402 = −0.02798 (OR = 0.972). The gender coefficient remains highly significant (*p*-value = 0.003), but its value decreased almost twice (from 0.5713 to 0.30535), thus indicating a lower direct influence of gender on the asthma probability. In this model, the influence of gender on disease is partly explained by different age dependency slopes for boys and girls.

We compared alternative models presented in Table 5 and Table 6 using the Akaike information criterion. A model containing an age and gender interaction is considered superior with respect to this criterion. 

We performed the Hosmer and Lemeshow goodness of fit test for the model with interaction. The test showed a good match (*p*-value = 0.5886) between the expected and observed values of disease rates.

## 4. Discussion

Our study is evaluating the complex impact of air pollution, proximity of green spaces, distance to traffic, age of children and gender characteristics. This study adds a piece of knowledge to the overall research highlighting the air pollution, green spaces and children’s asthma relationship. GIS makes it possible to combine the spatial location of the cases and controls, and to relate it to air pollution and green spaces maps.

Our data with links between asthma cases and NO_2_ pollution fall into a list of studies relating health outcomes with pollutants in the urban environment. Nitrogen dioxide is one of the most common atmospheric pollutants found in urban areas. It is a factor that causes irritation of the respiratory airways, bronchial sensitivity and lung function changes [29]. Experimental studies with human volunteers show lung immune responses and changes in airway hyperresponsiveness, even at concentrations below recommended levels [30]. Studies in Taiwan have found that a 10% increase in concentration caused the asthma outpatient visit rate to increase by 0.30% (95% confidence interval (CI): 0.16%~0.45%) [31]. A large study evaluating an annual worldwide number of children with asthma, states that asthma incidence occurred in areas with annual NO_2_ concentrations even lower than the World Health Organization limits [32]. At the same time, there are studies that do not confirm the relationship between nitrogen oxide emissions and asthma. The Southern California Children’s Health Study states that there were independent associations of children asthma with traffic-related pollution at school and home, whereas the estimate for NO_2_ was attenuated (HR 1.37; 95% CI, 0.69–2.71). A Norwegian study did not find positive associations of long-term traffic-related exposures with asthma onset for children in Oslo [33].

Our study also adds additional knowledge to investigations exploring the impact of different pollution exposure assessments. Past researchers evaluated annual doses of pollutants [34], and some other studies evaluated daily doses, adding a time lap for the assessment of the links between pollution and health [35]. In our opinion, it makes more sense to use annual pollution exposure and to relate it to annual morbidity data. Using day to day methods, there can be different gaps between illness and the time of diagnosis. This can lead to significant errors linking pollution and illness.

We used the ADMS model for pollution modeling. The relationship between the pollutants and their distance distribution from the roads and green spaces was linked with each other, showing that the chosen simulation method is sensitive. Road transport-emitted pollutants (NO_2_, CO, PM_10_, PM_2.5_, B[a]P) correlated respectively and strongly with each other, while the industry pollutant SO_2_ had a weak link to other pollutants and proximity to roads. The chosen ADMS is an advanced model for calculating concentrations using the Gaussian spreading algorithm of pollutants emitted both continuously from the line, point and area sources, widely used in other European countries [36]. The comparison of ADMS with real contaminant concentrations measured in London has demonstrated good performance of the model, with the annual mean values of PM_10_ (overall fractional bias 0.048) and NO_2_ (overall fractional bias 0.02) especially well predicted. Of course, there are many modeling and interpolation methods, but they do not provide significantly different estimates [37].

Our results indicated that living near green environments decreases the risk of asthma and is safer due to the lower pollution in the area. Evaluating reviewed studies, some of them reported no association between green spaces and child’s asthma, while another reported decreased morbidity due to asthma near green spaces [14]. The positive effect can be related to lower air pollution in the green spaces; the negative correlation can be related to the effect on asthma of pollen [15]. On the other side, outdoor activities required a higher volume of breathed air by inhaling more pollutants. Research findings are not sufficient to estimate the significance of the distance to green areas, but our study shows a weak positive influence. 

Our study of the 7–17-years-old child population results evaluating gender impact are very similar to other studies. According to health statistics, 2/3 of children with asthma were males and 1/3 were females: in our research, 64.1% with asthma were boys and 35.9% were girls. Increased risk of asthma in boys can be explained by immunological aspects, airway size and growth differences [6].

The very large sample size, individual data with annual air pollution level, the exact distance to green spaces and confirmed diagnoses of asthma are the strong points of our study. The use of ADMS modeling and multivariate analyses reveals the associations between exposure to air pollutants and children’s asthma morbidity. We assumed that children’s exposure occurs at their living place and the surrounding region.

Our study had some limitations. A cross-sectional study evaluates only the relationship between factors and morbidity. We had no other socioeconomic confounding variables to include in the analysis. This has interesting pros and cons for analysis. Cons: we do not have socioeconomic variables; pros: we can have a large dataset with more than 51,000 children included. This a complete set of all (selected age) children who live in Vilnius, Lithuania. We cannot have such a complete dataset using a survey. Further, I want to mention that environmental factors, both individually and in combination with genetic factors, are considered to be major determinants. Pollution data are modeled, the real-time measurements would contribute more accurate data, but this is impossible and expensive for a large population. We mentioned earlier that the pollution map model is validated and shows great prediction results. This is an important addition to the research assessing the risk of asthma and environmental factors in an urban area.

## 5. Conclusions

In conclusion, the Vilnius study provides evidence of an adverse effect on the risk of asthma for children living in an area with a higher concentration of NO_2_ and residence proximity from green spaces. The influence of gender on disease is partly explained by different age dependency slopes for boys and girls. The complex evaluation shows that boys and younger children have an increased risk of developing asthma. According to our study, reducing urban transport pollution may be one of the factors in reducing the incidence of bronchial asthma in children. Assuming that air pollution levels are linked to childhood asthma, efforts to reduce pollution could help prevent new cases of asthma.

## Figures and Tables

**Table 1:** Distribution of air pollution in 2017 (Vilnius).

Air Pollutants	Quality in 2017 Year (Data from 4 Air Quality Stations)				Air Quality Limits
	1	2	3	4	
PM_10_ (days)	30	9	3	3	(35 days) > 50 µg/m^3^
PM_10_ µg/m^3^ (annual average)	35	26	23	19	40 µg/m^3^
PM_2.5_ µg/m^3^ (annual average)	17	-	-	-	25 µg/m^3^
NO_2_ µg/m^3^ (annual average)	34	18	14	15	40 µg/m^3^
SO_2_ µg/m^3^ (annual average)	-	4.5	4	4.6	-
B[a]P ng/m^3^ (annual average)	1.14	-	-	-	1 ng/m^3^ (target value)

**Table 2:** Distribution of all children aged 7–17 years (N) and children of this age with asthma (n) according to gender and age.

Age	*N* (All Children)						*n* (Children with Asthma)					
	Boys		Girls		All		Boys		Girls		All	
	Count	%	Count	%	Count	%	Count	%	Count	%	Count	%
7	3209	6.3	3153	6.2	6362	12.4	255	8.3	161	5.3	416	13.6
8	3097	6.0	2922	5.7	6019	11.7	238	7.8	149	4.9	387	12.6
9	2871	5.6	2737	5.3	5608	10.9	246	8.0	138	4.5	384	12.5
10	2501	4.9	2393	4.7	4894	9.6	206	6.7	117	3.8	323	10.5
11	2363	4.6	2326	4.5	4689	9.2	177	5.8	100	3.3	277	9.0
12	2150	4.2	2072	4.0	4222	8.2	174	5.7	113	3.7	287	9.4
13	2109	4.1	1988	3.9	4097	8.0	151	4.9	73	2.4	224	7.3
14	1988	3.9	1848	3.6	3836	7.5	140	4.6	55	1.8	195	6.4
15	1881	3.7	1804	3.5	3685	7.2	125	4.1	62	2.0	187	6.1
16	1934	3.8	1870	3.6	3804	7.4	126	4.1	65	2.1	191	6.2
17	2058	4.0	1971	3.8	4029	7.9	126	4.1	68	2.2	194	6.3
All	26,161	51	25,084	49	51,245	100.0	1964	64.1	1101	35.9	3065	100

**Table 3:** Spearman correlation matrix for air pollutants and proximity from the main roads and green spaces.

	PM_10_	PM_2.5_	NO_2_	SO_2_	CO	B[a]P
PM_10_		0.97	0.51	0.17	0.53	0.74
PM_2.5_	0.97		0.51	0.17	0.53	0.74
NO_2_	0.51	0.51		0.19	0.85	0.73
SO_2_	0.17	0.17	0.19		0.68	0.22
CO	0.53	0.53	0.85	0.68		0.65
B[a]P	0.74	0.74	0.73	0.22	0.65	
Proximity to Intensive Roads	−0.18	−0.18	−0.28	−0.19	−0.30	−0.29
Proximity to Green Spaces	0.24	0.23	0.22	0.13	0.14	0.28

**Table 4:** The odds ratio of asthma in relation to the age of children, gender and air pollutants (simple logistic regression analysis performed for every variable separately).

	β coef.	OR	CI 95%	*p*-Value
Age	−0.03623	0.964	(0.953 to 0.975)	<0.001 *
Gender (male = 1)	0.56988	1.768	(1.639 to 1.907)	<0.001 *
PM_10_	0.01198	1.012	(1.003 to 1.021)	0.008 *
PM_2.5_	0.03655	1.037	(1.010 to 1.064)	0.006 *
SO_2_	0.07612	1.079	(0.984 to 1.184)	0.107
NO_2_	0.01635	1.165	(1.005 to 1.027)	0.003 *
CO	0.44336	1.558	(0.687 to 3.519)	0.287
B[a]P	0.21984	1.246	(1075 to 1.440)	0.003 *
Proximity to Roads	−0.10100	0.904	(0.723 to 1.123)	0.368
Proximity to Green Spaces	0.33120	1.393	(1.121 to 1.742)	0.002 *

β coef.—coefficient estimated by the logistic regression; OR—Odds ratio; CI—Confidence interval * *p* < 0.05.

**Table 5:** The odds ratio of asthma in relation to the age of children, gender and air pollutants (multiple logistic regression model performed for variables significant in simple logistic regression model, see Table 4, with stepwise variable selection applied).

	β coef.	OR	CI 95%	*p*-Value
Age	−0.03672	0.964	(0.953 to 0.975)	<0.001 *
Gender (male = 1)	0.57130	1.770	(1.641 to 1.911)	<0.001 *
NO_2_	0.01347	1.013	(1.002 to 1.025)	0.018 *
Proximity to Green Spaces	0.22038	1.336	(1.060 to 1.653)	0.013 *
PM_10_	0.00015	1.0002	(0.998 to 1.002)	0.860
PM_2.5_	0.00012	1.0001	(0.998 to 1.003)	0.925
B[a]P	0.00620	1.006	(0.955 to 1.059)	0.815

β coef.—coefficient estimated by the logistic regression; * *p* < 0.05.

**Table 6:** The odds ratio of asthma in relation to the age of children, gender, air pollutants and their pairwise interactions (multiple logistic regression model performed for variables significant in simple logistic regression model, see Table 4, with stepwise variable selection applied).

	β coef.	OR	CI 95%	*p*-Value
Age	−0.05200	0.949	(0.931 to 0.968)	<0.001 *
Gender (male = 1)	0.30535	1.357	(1.029 to 1.791)	0.003 *
NO_2_	0.01340	1.013	(1.002 to 1.025)	0.019 *
Proximity to Green Spaces	0.28264	1.327	(1.061 to 1.654)	0.013 *
Age × Gender (male = 1)	0.02402	1.024	(1.000 to 1.050)	0.051

β coef.—coefficient estimated by the logistic regression; * *p* < 0.05.
